# Supplementary material for: Urban Air Pollution Particulates Suppress Human T-Cell Responses to Mycobacterium Tuberculosis
Source: Int J Environ Res Public Health. 2019 Oct 25;16(21):4112. doi: 10.3390/ijerph16214112 (PMC6862251; doi:10.3390/ijerph16214112)
Supplement: Supplementary file 1 [file ijerph-16-04112-s001.pdf]

Urban Air Pollution Particulates Suppress Human T-cell Responses to *Mycobacterium tuberculosis*

Olufunmilola Ibironke[1]; Claudia Carranza[2]; Srijata Sarkar[3], Martha Torres[2], Hyejeong Theresa Choi[4], Joyce Nwoko[3], Kathleen Black[4], Raul Quintana-Belmares[5], Álvaro Osornio-Vargas[6], Pamela Ohman-Strickland[7] and Stephan Schwander[3, 4,*]

*Corresponding author

Rutgers School of Public Health, Departments of Urban-Global Public Health and Environmental and Occupational Health, 1 Riverfront Plaza, Newark, NJ 07102, schwansk@sph.rutgers.edu, phone: 732-235-5405, Fax: 973-972-8032

Online Supplementary Figures

Figure S1. Effects of $PM_{2.5}$ on apoptosis of mononuclear cells

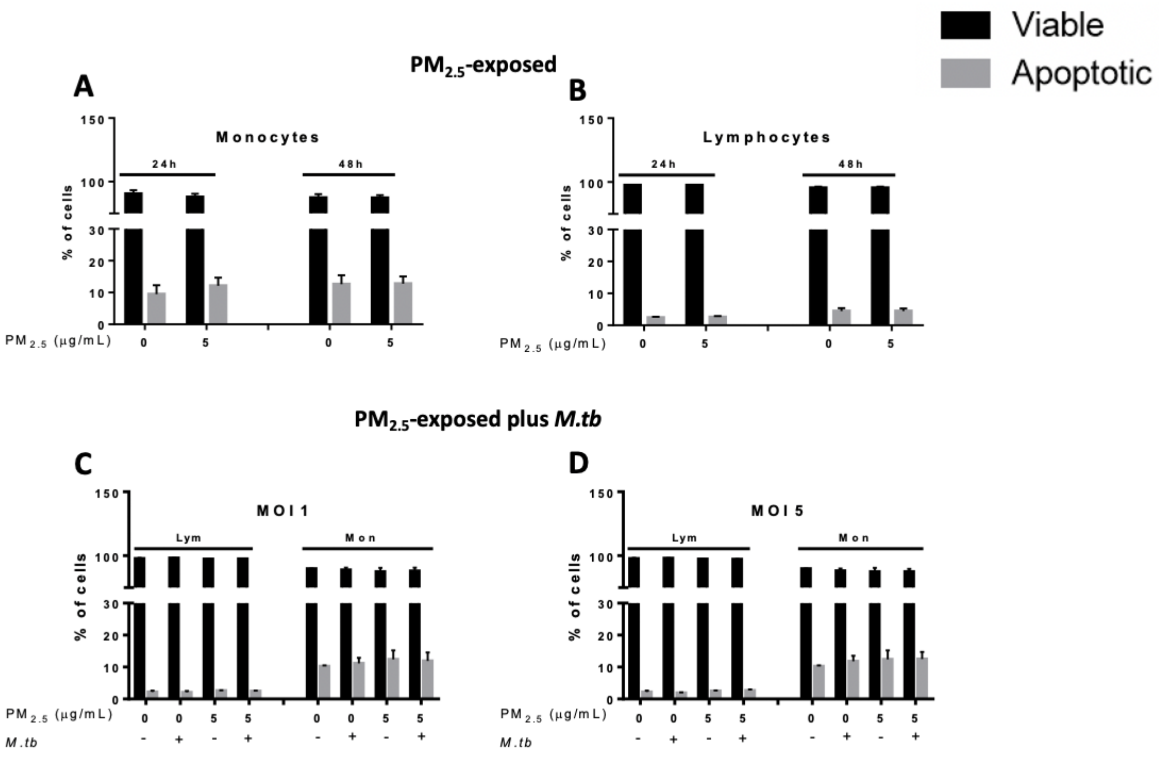

(A, B) PBMC from three study subjects were pre-exposed to $PM_{2.5}$ (5 μg/ml) for 24 and 48 h. Detection of phosphatidylserine exposure by Annexin-V FITC/propidium iodide double-staining was performed to assess the proportion of mononuclear cells that were undergoing apoptosis (Annexin V and Propidium Iodide positive). (C, D) $PM_{2.5}$ effects on apoptosis in PBMC from three study subjects pre-exposed to $PM_{2.5}$ for 20 h followed by infection with *M.tb* MOI 1 and 5 for 18 h were determined by detecting phosphatidylserine. Values are expressed as the mean +/- SEMs of three independent experiments (A, B, C, D).

Figure S2. Hypothetical model of PM-induced suppression of T cell immune responses to *M.tb*.

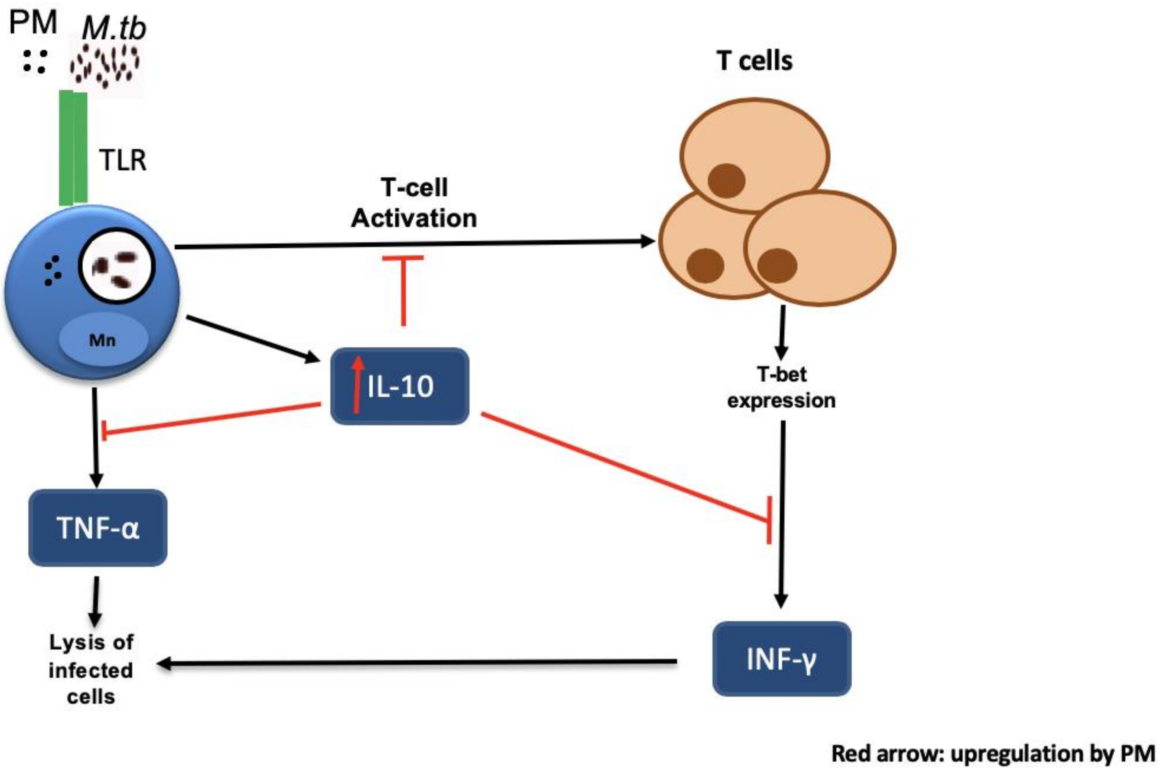

A prerequisite for T cell immune responses to *M.tb* infection, is the interaction of *M.tb* with toll like receptors (TLR) on monocytes resulting in their activation. Activated monocytes produce effector molecules and present antigens to naïve T cells resulting in the activation of the T cells and expression of transcription factor T-bet that positively regulates IFN-γ production. Activated T cells and monocytes produce several protective cytokines (including IFN-γ and TNF-α respectively) that can result in the killing of *M.tb*-infected cells. Air pollution PM exposure impairs antimycobacterial T cell immune responses by downregulating T cell activation, inhibiting the growth control of *M.tb* in infected cells, by impairing the production of protective cytokines (IFN-γ and TNF-α) and by augmenting the production of anti-inflammatory cytokine IL-10 as well as via downregulation of T-bet. Activation processes are shown with pointed black arrows, PM-induced upregulation of IL-10 is shown with an upward-pointing red arrow, while PM-induced inhibition via IL-10 is shown by blunt-end red arrows. (TLR, toll like receptors; Mn, monocyte).
